# “Where Are You Really From?”: The Blurred Fences of the Race and Ethnicity Data Box

**DOI:** 10.1002/nop2.70230

**Published:** 2025-04-17

**Authors:** Della Maneze, Binu Koirala, Brandon W. Smith, Diana‐lyn Baptiste, Gemma McErlean, Lucie M. Ramjan

**Affiliations:** ^1^ School of Nursing, Faculty of Science, Medicine and Health University of Wollongong Wollongong New South Wales Australia; ^2^ School of Nursing and Midwifery Western Sydney University Parramatta New South Wales Australia; ^3^ School of Nursing Johns Hopkins University Baltimore Maryland USA; ^4^ Centre for Research in Nursing and Health St. George Hospital Sydney New South Wales Australia

Imagine for a moment you are a researcher collecting data. You receive the following sociodemographic profile for a participant:

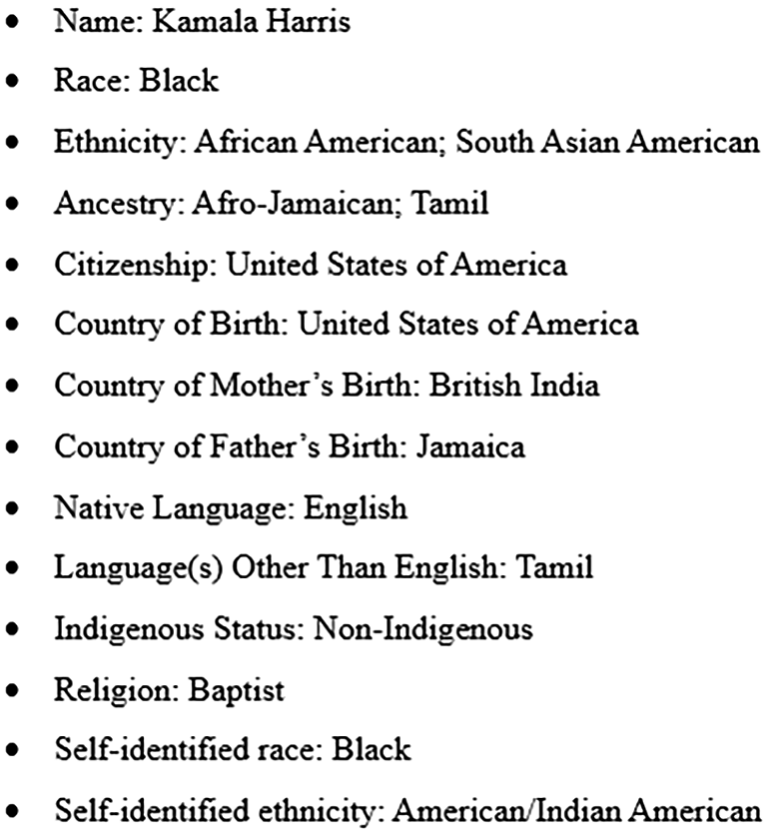

“Where are you really from?” is a question often asked when a person's name, appearance, or accent is different, or does not align with someone's expectations. While the question may seem straightforward, in the context of race and ethnicity data collection, this is not an easy question to answer. Take, for example, former United States Vice President Kamala Harris, a person of both Indian and Jamaican descent—“where are you from?” is a much more layered and multifaceted question than it may initially seem.

During the 2024 presidential campaign, Kamala Harris' story was one of contested identity and intense scrutiny (Ma et al., Ma et al. 2021). While the campaign's discourse is beyond the scope of this discussion, the controversy surrounding her heritage brings to light a broader issue in contemporary research: how to collect, interpret, contextualise, and present demographic data in a way that captures its inherent complexity while respecting the narrative truth of individual identities.

The reporting of race and ethnicity in research can be a contentious issue warranting some caution, primarily because “the concepts are dynamic, highly contextual, and multidimensional, incorporating social, political, and geographical factors” (National Academies of Sciences, 2024). Biomedical research can be framed as asking “what are you?”, focusing on static variables including biological or genetic attributes. Conversely, the question “who are you?” in social science research, can be about exploring the dynamic variables related to sociocultural identities and lived experiences. The dichotomy between these approaches reflects a fundamental tension: how do we balance scientific rigour with the diversity of human identity?

*Pragmatic* guidelines currently offer directions on the collection of this data in biomedical research from the point of study conception through to publication (Lewis et al. 2023), with a fervent push towards community engagement and rigorous transparency in how and why this data is being collected and reported. These efforts are intended to mitigate bias, foster inclusivity and community trust, and enhance a meaningful understanding of health disparities (Alvidrez et al. 2019; National Academies of Sciences, 2024). A fundamental challenge is the inherent fluidity and dynamism of ethnic and racial categories, which are constantly evolving and shifting. The notion of a fixed, monolithic identity is a flawed construct, as individuals may identify with multiple ethnic or cultural affiliations, or their sense of identity may change over time. For example, immigration, cross‐cultural child adoption, or interracial marriages are among social changes that could impact ethnicity identification. This raises questions about the adequacy of relying on static categories or self‐reported data examined in isolation to capture the rich diversity within and across communities while concurrently finding a balance to avoid the potential reinforcement of stereotypes, misinterpretation of data and overlooking critical social determinants of health.

Current systems of collecting data on race, ethnicity, and culture are limited by oversimplifying populations and aggregating them into broad categories. This reductionist lens of traditional population descriptors or labels, such as ‘White’, ‘Black’ or ‘Asian’ fail to authentically capture the complexities and nuances of lived experiences, the fluidity of identities, and the social determinants that shape health (Alvidrez et al. 2019). A ubiquitous example of combining populations is in the ‘Asian’ category which encompasses a wide range of genetically distinct and culturally diverse groups from 48 countries. Furthermore, the use of the term “Asian” ethnicity typically refers to the geographic origin of a population from the Asian continent, which has been shown to have substantial population substructures at a subcontinental level (National Academies of Sciences, 2024). Similarly, in the United States, the term ‘African American’ is commonly used to group all Black individuals, assuming that everyone of African descent fits into this category. However, the Black community is diverse and not monolithic. There are significant differences among subgroups within the African diaspora, including variations in culture, language, societal norms, and health risks (Agyemang et al. 2005). Therefore, combining these groups can introduce substantial errors and bias into study designs at all levels, be it sociocultural, environmental and genetics, obscuring important differences in health risks and outcomes due to the extensive heterogeneity of “subpopulation” group categories (Alvidrez et al. 2019; National Academies of Sciences, 2024). This might have been reasonable in the past because ethnic minority populations were often underrepresented in research due to smaller sample sizes, making it challenging to draw meaningful conclusions or identify specific health disparities within these groups. However, with time, there is a need to reassess these classification systems and update how we define, collect, and interpret race, culture, and ethnicity data which should reflect the diversity of the current population affecting policies and outcomes. For instance, how would it be possible for Kamala Harris to choose her identity in biomedical research as someone who is multiracial and multiethnic? Is she forced to choose the ‘best’ fit? Or if multiracial and multiethnic are single categories then could we not all fit into one of those profiles?

Given race and ethnicity are socially constructed rather than biologically determined, a standardised approach for their conceptualisation is crucial and should be complemented by the option to self‐identify, capturing diversity within a population and the nuance of lived individual human experiences, thereby reducing racial and ethnic disparities in research, enhancing accuracy in identifying racial and ethnic identities (National Academies of Sciences, 2024). This evolving process requires not only capturing biological and cultural factors but also recognising the role of socioeconomic and environmental influences in redefining these variables. Consistency and clarity in the reporting of race and ethnicity are essential for making meaningful comparisons across studies and in developing targeted interventions. This will allow for more precise identification of risk factors, and ultimately, more effective health prevention and management strategies that address the unique needs of diverse populations. Importantly, the aims of the research and the relevance of collecting race and ethnicity data to the research must also be considered critically to ensure that the use of these concepts is fit for purpose.

Despite challenges, determining sociocultural identification—whether through race or ethnicity—is critical in health research because it helps to capture the complex interplay of biological, social, cultural and environmental factors that affect health risks and outcomes. While race itself may not be a direct cause of disease, it often serves as a proxy for underlying socioeconomic, cultural and access‐related factors that can significantly influence health. By accurately understanding and accounting for racial and ethnic identification, health researchers can better assess how these factors contribute to health disparities, particularly in minority populations.

Although defining categories of race and ethnicity can arguably be complex and challenging, some recommendations for moving towards greater transparency and clarifying the “fences” of race and ethnicity data collection include:

1. *Justification and transparency*: Clearly define the rationale behind collecting race and ethnicity data, ensuring transparency in methodologies, classifications, criteria and potential limitations.
2. *Nuanced categorisation and contextualised data*: Provide operational definitions of race and ethnicity categories avoiding overly broad classifications that homogenise and erase complex histories, identities, and lived experiences. Race and ethnicity data do not exist in isolation and should be recognised as intersecting with relevant social determinants of health such as socioeconomic status, education, and environmental factors. Hence, data collection should account for this intersectionality.
3. *Flexible models*: Recognise that identity is fluid and multidimensional; there is no universal measurement that accurately captures its complexity. Allow for multiple selections and self‐identification to better reflect personal, ancestral, and experiential realities. While this may complicate data analysis, this model could be more accurate in capturing the visible and invisible facets of identity.
4. *Community engagement and codesign*: Establish meaningful partnerships with affected communities, actively involving them in critical stages of research design and implementation, to promote cultural sensitivity, reduce bias and prevent misrepresentation or misapplication of findings.
5. *Review and update methods*: The approach to data collection and interpretation for race and ethnicity data should be reviewed periodically to ensure it reflects current societal trends, addresses emerging issues, and incorporates feedback from underserved communities.

The identification of race and ethnicity will continue to be a subject for debate, yet these remain important demographic variables routinely collected in both social and biomedical research. However, as Kamala Harris exemplifies, the future of race and ethnicity data collection should dispute the assumption that a single label captures a person's full story. It is critical for health researchers to acknowledge and understand the diversity within racial groups, particularly those with complex genetic backgrounds and varied origins. This understanding is essential for healthcare systems to effectively address health disparities and strive for greater equity among minority populations whose ethnicities are often generalised into broad categories. Research must shift towards frameworks that honour the full spectrum of human identity, ensuring race and ethnicity categories are as diverse as the people they seek to represent.

## Conflicts of Interest

The authors declare no conflicts of interest.

## Data Availability

The authors have nothing to report.
